# Insight into the integration effect of chitosan and β-cyclodextrin on the properties of zinc-phosphate/hydroxyapatite hybrid as delivery structures for 5-fluorouracil: loading and release profiles

**DOI:** 10.3389/fchem.2024.1456057

**Published:** 2024-09-11

**Authors:** May N. Bin Jumah, Sarah I. Al Othman, Awatif Abdulaziz Alomari, Ahmed A. Allam, Stefano Bellucci, Mostafa R. Abukhadra

**Affiliations:** ^1^ Biology Department, College of Science, Princess Nourah bint Abdulrahman University, Riyadh, Saudi Arabia; ^2^ Zoology Department, Faculty of Science, Beni-Suef University, Beni-Suef, Egypt; ^3^ Department of Biology, College of Science, Imam Mohammad Ibn Saud Islamic University (IMSIU), Riyadh, Saudi Arabia; ^4^ INFN-Laboratori Nazionali di Frascati, Frascati, Italy; ^5^ Geology Department, Faculty of Science, Beni-Suef University, Beni-Suef, Egypt; ^6^ Materials Technologies and their Applications Lab, Geology Department, Faculty of Science, Beni-Suef University, Beni-Suef City, Egypt

**Keywords:** hydroxyapatite, biopolymers, composites, 5-fluorouracil, loading, anticancer

## Abstract

Zinc-phosphate/hydroxyapatite hybrid form (ZP/HP) in core-shell nanostructure was developed and functionalized with both chitosan (CS@ZP/HP) and β-cyclodextrin (CD@ZP/HP) as bio-composite of enhanced physicochemical and biological properties. These structures were assessed as potential deliveries of 5-fluorouracil, exhibiting enhanced loading, release, and anti-cancer behaviors. The functionalization strongly prompted the loading effectiveness to be 301.3 mg/g (CS@ZP/HP) and 342.8 mg/g (CD@ZP/HP) instead of 238.9 mg/g for ZP/HP. The loading activities were assessed based on the hypotheses of traditional kinetic and isotherm models, alongside the computational variables of the monolayer model with a single energetic site as an advanced isotherm model. The functionalized versions exhibit much greater loading efficacy compared to ZP/HP as a result of the increment in the density of the existing loading sites [Nm_(5-Fu)_ = 78.85 mg/g (ZP/HP), 93.87 mg/g (CS@ZP/HP), and 117.8 mg/g (CD@ZP/HP)]. Furthermore, the loading energies of approximately 40 kJ/mol, together with the loading potential of each receptor (n > 1) and Gaussian energies of approximately 8 kJ/mol, indicate the physical entrapment of 5-Fu molecules according to a vertical orientation. The materials mentioned verify long-term and continuous release characteristics. Following the modification processes, this behavior became faster as both CS@ZP/HP and CD@ZP/HP displayed complete release within 120 h at pH 1.2. The kinetic studies and diffusing exponent (>0.45) indicate that release characteristics are controlled by both diffusion and erosion processes. These carriers also markedly increase the cytotoxicity of 5-Fu against HCT-116 colorectal cancer cell lines: 5-Fu-ZP/HP (3.2% cell viability), 5-Fu-CS@ZP/HP (1.12% cell viability), and 5-Fu-CD@ZP/HP (0.63% cell viability).

## 1 Introduction

Approximately 72% of all recorded deaths worldwide were attributed to non-communicable diseases, mostly cancer, and this proportion is projected to rise towards 75% within the coming years (El-Zeiny et al., 2020; Cutrim et al., 2021). Colorectal carcinoma is a common and aggressive form of cancer that impacts roughly 13% of people suffering from cancer worldwide. It represents one of the most common reasons for death, contributing to the overall international fatality rate (Abuzar et al., 2020; Othman et al., 2021; Tian et al., 2020). Colorectal carcinoma originates as a polyp within the mucous tissues and spreads to invade the submucosa along with adjacent tissues. During the more advanced stages of colorectal carcinoma, the tumor cells formed penetrate the nearby tissues alongside lymph nodes (Sundaramoorthy et al., 2016). Consequently, the top priority for the healthcare and scientific sectors is to produce effective and safe medicines that may suppress malignant cells without triggering any significant adverse effects (Tian et al., 2020; Praphakar et al., 2018).

Various chemotherapy medications are often used to suppress the development and replication of cancerous cells (Lee J. E. et al., 2019; Itoo et al., 2022). Commonly administered chemotherapies generate a significant level of oxidative stress and effectively hinder the mechanism of DNA reproduction, leading to the destruction of malignant cells (Tian et al., 2020; Li et al., 2021). Regrettably, almost all of the chemotherapy medications now in use possess toxic consequences for healthy tissue and result in many kinds of serious adverse effects, especially if administered in elevated doses. These negative effects involve nausea, renal damage, and impairment of the bone marrow’s function. As a result, much research was conducted to improve the safety, compatibility with living organisms, therapeutic characteristics, and targeted effects of existing traditional chemotherapy agents (Sayed et al., 2022a). It was suggested that this improvement could be achieved by either developing new anticancer medications or by strengthening the biological safety and efficacy of commonly existing traditional types (Abuzar et al., 2020).

5-Fluorouracil (5-Fu) is an extensively utilized chemotherapeutic agent for treating several types of malignancy cells, particularly rectum, breasts, colorectal, and gastric malignancies (El-Zeiny et al., 2020; Tan et al., 2018). Regrettably, similar to the other types of chemotherapy medications, the use of 5-Fu is linked to various negative aspects resulting from its restricted solubility, low specificity, and rapid release speed. Furthermore, there are numerous reports of serious toxicity resulting from overdoses of 5-Fu (Abukhadra et al., 2019; Çiftçi et al., 2020). 5-Fu demonstrates strong toxicity towards the neurological, digestive, hematological, cardiac, hematological, and dermatological systems (Tan et al., 2018; Lee et al., 2019b). Consequently, many innovative systems for administration were examined as efficient strategies to enhance the therapeutic activities, curative significance, solubility, releasing speed, and specificity of 5-Fu (El-Zeiny et al., 2020; Sayed et al., 2022a; Vatanparast and Shariatinia, 2018). It is highly encouraged to effectively load drugs into improved carriers that are compatible with the human system. This will assist in controlling the quantity being administered and ensuring it is delivered during particular periods and rates. As a result, it may minimize the most frequently described adverse effects and prolong the time frame of contact (Shad et al., 2020; Luo et al., 2016; Khang et al., 2020). In addition, this has the potential to greatly improve patient compliance and therapeutic characteristics while also minimizing the speed at which the medication degrades, maintaining the amount administered at the specified level (Luo et al., 2016; Khang et al., 2020).

Zeolite, polymers, mesoporous silica, CaO, layered double hydroxide (LDH), montmorillonite, and hydroxyapatite, along with various inorganic as well as organic compounds, were all evaluated as possible carriers for traditional chemotherapy medications (Othman et al., 2021; Tian et al., 2020; Sayed et al., 2022a; Abukhadra et al., 2020; Rahbar et al., 2020). The drug’s permeability and retention qualities were significantly improved by applying the prior materials as delivery structures (Othman et al., 2021; Tian et al., 2020; Sundaramoorthy et al., 2016; Itoo et al., 2022; Shad et al., 2020). Different types of hydroxyapatite (HAP), including its mixed and composite frameworks were examined as transporters for a variety of chemotherapy medications. HAP comprises a widely recognized commercial biomaterial that belongs to the apatite group and possesses a chemical composition of Ca_10_(PO_4_)_6_(OH)_2_. Moreover, it is extensively employed in a number of medical fields, including tissue and bone engineering, and additionally for administering medications due to its remarkable effectiveness in loading and releasing (Barbosa et al., 2020; Veerla et al., 2019; Cai et al., 2020; Tran et al., 2022). The technological and biological value of HAP could be ascribed to its significant bioselectivity, prolonged shelf life, extensive surface area, chemical versatility, impressive ion exchange efficiency, adaptable framework, and strong adsorption efficacy (Ofudje et al., 2021; Pai et al., 2022; Chaudhry et al., 2022). Being a biomaterial, it has the potential to be utilized with live tissues, possesses bioactive qualities, degrades spontaneously, and promotes bone growth without injuring or inflaming surrounding tissue (Lee et al., 2019a; Pujari-Palmer et al., 2016). Furthermore, earlier studies demonstrated its significant inhibitory effect on specific tumor cells, such as MGC-803, breasts, Bel-7402, and Os-732 (Seyfoori et al., 2019). However, the hydrophilic properties of the crystalline form reduce its efficacy as a carrier for traditional medications with an organic molecular structure (Huang et al., 2022; Tran et al., 2022). Moreover, the HAP nanomaterials demonstrate inadequate stability whenever subjected to pH-acidic situations, which include those detected in the gastrointestinal tract. This attribute impedes their appropriateness for administration via mouth (Munir et al., 2021).

As a result, several studies have been conducted to improve the biological, chemical, and physical properties of HAP through modification of its shape, chemical composition, and crystal size. Furthermore, other studies have explored the modifications of the exterior surface by organic groups and the incorporation of HAP into composites with a variety of polymers in conjunction with other chemically active ingredients (Huang et al., 2022; Wan et al., 2017; Fan et al., 2021; Mossavi et al., 2022; Elsayed et al., 2023). The HAP nanomaterials, which had been modified chemically using carboxylic groups, had greater inhibition on the proliferation of cancerous cells compared to the unmodified nanoparticles. Moreover, the blended products have the capacity to control the release of medicines from the HAP structure because of the existence of carboxylic functions, which improve the binding of drug molecules to its interface (Lee et al., 2019b).

β-cyclodextrin (β-CD) is an extensively examined and highly appreciated biopolymer that is frequently incorporated as a key element in several blends containing various inorganic chemicals. Composite materials have numerous applications in the sectors of medical and environmental safety (Saad et al., 2020; Sadjadi and Koohestani, 2021). The main reasons for this were primarily its non-toxic nature, being readily available, strong resistance to chemicals, high effectiveness in adsorption, and compatibility with biological systems (El-Sherbeeny et al., 2021; Krawczyk et al., 2022). β-CD consists of a cyclic structure made up of 6–7 glucose units that are connected through α (1→4) glycosidic linkages (Sadjadi and Koohestani, 2021; Bandura et al., 2021). The internal surfaces of β-CD exhibit hydrophobic properties; however, the external interfaces of such units and their functional chemical contents display significant polarity (Jumah et al., 2021). This significantly increases its integration into composites involving many types of inorganic components and enhances the drug-loading characteristics of synthetic carriers (El-Sherbeeny et al., 2021; Jiang et al., 2020). Furthermore, β-CD functions as a carrier or vehicle for administering medications and has a significant influence on the chemistry and physics of the encapsulated medications. This includes its soluble state, therapeutic advantages, as well as the long-term stability of the drug’s chemical structure and physical attributes (Wang et al., 2023). Also, chitosan, a well-recognized biopolymer, performs a vital role in several fields such as pharmacology, environmental science, and medicine. It is also extensively utilized as a carrier for pharmaceutical products (Saad et al., 2020; Jiang et al., 2020; Ibrahim et al., 2021). It is a polysaccharide complex produced from chitin, which is found in several naturally derived supplies (Saad et al., 2020). In addition to having advantageous mechanical and adsorption qualities, chitosan chains are known for their security, hemostatic efficacy, biological activity, antimicrobial potential, biocompatibility, and biodegrading qualities (Jiang et al., 2020).

This work seeks to investigate the effects of surface modifications on synthesized zinc phosphate/hydroxyapatite nanostructures using chitosan (CS@ZP/HP) and β-cyclodextrin (CD@ZP/HP) biopolymers to enhance the characteristics of the resulting bio-composites, which will be used as carriers for the medication 5-fluorouracil. The delivery characteristics of the composites were evaluated by a comprehensive analysis of their loading aspects and the mechanisms that influence their performance. The investigation also included a presentation and discussion of the release’s characteristics and releasing kinetics of the materials tested, as well as their cytotoxicity functions as chemotherapy agents towards colorectal cancer cells (HCT-116).

## 2 Experimental work

### 2.1 Materials

The starting materials of magnesium and calcium have been extracted from raw Egyptian limestone and applied across the various stages of developing apatite-based nanomaterials. The chemicals employed during the dissolving and development of crystals processes include nitric acid (40%), zinc chloride (with a purity of over 98.0%), phosphoric acid (88%), and ammonia solution (containing 25% NH_3_). The hydroxyapatite hybridization technique involved using highly pure β-cyclodextrin (>85%; MW 1,153 g/mol) and pure ethanol (95%) supplied by Sigma-Aldrich, Egypt. In order to synthesize a chitosan-based hybrid, acetic acid with a quality of 99.8% and de-acetylated chitosan particles with a MW of 120,000 were applied. 5-fluorouracil (>99%) has been employed to perform the loading process, releasing, and cytotoxic investigations.

### 2.2 Synthesis of ZP/HP structure

The limestone fragments were ground inside a ball mill, producing particulates with an average size that varied from 10 to 150 μm. The powdered limestone, approximately 35 g, was immersed within 150 mL of 40% HNO_3_ employing a magnetic stirring device (500 rpm) at room temperature over 48 h as long as it was completely dissolved. A 45-µm Whatman filtering paper was used to separate the solid residues from a passed solution, which consists of a high concentration of soluble Ca^2+^ and Mg^2+^ ions. The solution that had been obtained, with a volume of 150 mL, was subsequently mixed with 10 g of zinc chloride and agitated for a duration of 5 h until their full dissolution was attained. Afterwards, a certain amount of phosphoric acid (150 mL), which acts as the supplier of PO_4_^3−^, was added to the resulting mixture while stirring continuously for a period of 2 h. The subsequent phase in the course of production of HAP included a fast precipitation process, which involved stirring the resultant mixture and progressively introducing NH_4_OH while being subjected to 240 W of ultrasonic waves for a duration of 60 min. The collected particles have been rinsed with distilled water over 15 min to attain neutralization. Following that, they were transferred to an electric dryer machine and exposed to a temperature of approximately 100°C over a duration of 24 h. The structure that developed was identified as ZP/HP and was kept for use in further characterization and more investigations.

### 2.3 Synthesis of CD@ZP/HP composite

The process of modifying ZP/HP nanomaterials with β-cyclodextrin was conducted following the method outlined by (Altoom et al., 2022). The β-CD particles (1 g) were first dispersed and mixed within about 80 mL of ethanol using a magnetic stirrer working at 1,000 rpm over 3 h until a slurry-like solution was formed. In an independent experiment, 2 g of produced ZP/HP particles were uniformly dispersed and homogenized by spinning them at 1,000 rpm inside 100 mL of distillate water for 60 min, supplemented by a 240-watt ultrasonic generator. Afterwards, the ZP/HP slurry and β-CD mixtures were integrated and blended extensively by agitating at a velocity of 1,000 rpm for 24 h. Following that, a further mixing process was conducted for an extra 24 h using the ultrasound generator. Subsequently, the blended nanoparticles that had developed were extracted from the residual solutions by filtering them using a Whatman filter paper with a pore diameter of 40 µm. The resulting product was washed with distilled water to neutralize the surface of the CD@ZP/HP hybrid and subsequently dried for 12 h at 60°C.

### 2.4 Synthesis of CS@ZP/HP composite

After synthesizing the ZP/HP framework, a uniform slurry was obtained by pulverizing 2 g of the formed particles through 50 mL of distillate water before subjecting the obtained blend to ultrasound treatment at 240 watts over 120 min. A quantity of 4 g of grounded chitosan was submerged in 50 mL of acetic acid at a concentration of 0.1%. This solution was prepared separately and then carefully introduced to the previously prepared ZP/HAP mixture. The solution was agitated using a magnetic stirrer at a speed of 800 rpm for a duration of 24 h while simultaneously being exposed to 240 watts of ultrasonic waves. After undergoing thorough rinsing, the resultant material underwent filtering to eliminate any remaining acid and was then dried in an oven at 60°C overnight.

### 2.5 Characterization techniques

The X-ray diffraction (XRD) patterns of ZP/HP, CS@ZP/HP, and CD@ZP/HP have been determined using a PANalytical Empyrean diffractometer. To analyze the crystallographic elements and the structural specifications of the evaluated materials, the scanning rate has been set at 5°/min, and the working voltage has been set at 40 kV. The evaluation of chemical changes was conducted through the analysis of the FT-IR spectra. The spectrum was obtained using a Fourier transform infrared spectrometer (FTIR-8400S; Shimadzu) that operates within the frequency range between 400 and 4,000 cm^−1^. The study of morphological modifications entailed analyzing photographs obtained with a Zeiss Ultra 55 Gemini scanning electron microscope. The materials were covered with a thin film of gold, and the accelerating voltage has been tested throughout an evaluation range of 5–30 kV. The BET and BJH procedures were used in the investigation to determine the distributions of pore sizes and specific surface area, respectively, using the Beckman Coulter SA3100 surface area analyzer.

### 2.6 5-Fu loading studies

The study evaluated the incorporation of 5-Fu inside ZP/HP, CS@ZP/HP, and CD@ZP/HP through a comprehensive investigation. The main focus was to determine the optimal dosage and maximum capacity for the encapsulated 5-Fu. The investigation tested the key factors of pH (ranging from 3 to 10), encapsulation duration (ranging from 1 to 18 h), 5-Fu content (ranging from 50 to 400 mg/L), and temperature (ranging from 20°C to 60°C). The ZP/HP, CS@ZP/HP, and CD@ZP/HP particulates had been effectively mixed within the 5-Fu solutions (50 mL) using a vortex rotator. The ZP/HP, CS@ZP/HP, and CD@ZP/HP particulates were eliminated from the 5-Fu solutions through filtration immediately after each test’s equilibrium period. The rest of the 5-Fu levels were subsequently measured employing a UV-Vis spectrophotometer at an adapted wavelength (λ_(max)_ = 266 nm). The leftover levels of 5-Fu were determined and used to calculate the loading capacities of ZP/HP, CS@ZP/HP, and CD@ZP/HP using Equation 1. The loading tests of 5-FU through ZP/HP, CS@ZP/HP, and CD@ZP/HP were conducted three times, and the resulting mean values, together with their corresponding standard deviations of 3.4%, have been considered.

$$\text{Loaded drug }\left(\text{mg}/g\right)=\frac{\left(\text{Initial}\;\text{concentration}-\text{Residual}\;\text{concentration}\right)\times \text{solvent}\;\text{volume}}{\text{Carrier}\;\text{weight}}$$ (1)

### 2.7 The release studies

The diffusion behaviors of 5-Fu from ZP/HP, CS@ZP/HP, and CD@ZP/HP composites were evaluated using two distinct biochemical buffers (gastric fluid, pH 1.2, and intestinal fluid, pH 7.4) at a temperature of 37.5 C. The ZP/HP, CS@ZP/HAP, and CD@ZP/HAP particulates preloaded with 5-Fu (100 mg/g) were distributed uniformly separately within 500 mL of the diffusion buffers being tested. The DISTEK dissolving devices were used to uniformly distribute the 5-Fu-loaded ZP/HP, CS@ZP/HAP, and CD@ZP/HAP particulates through the two different buffering agents for a duration of 180 h. The homogenization process was carried out at a rotating speed of 200 rpm. A UV-Vis spectrophotometer has been utilized to assess extracts of two separate buffering solutions (5 mL) picked up regularly from the bulk fluids to track the diffusing % of 5-Fu out of ZP/HP, CS@ZP/HP, and CD@ZP/HP. Throughout the duration of the release, the volumes had been maintained at precisely the same values by promptly replenishing the bulk release buffers with the periodically extracted samples. The 5-Fu releasing tests were conducted three times, and the mean findings were obtained by applying Equation 2. The standard deviation was identified as being less than 3.75%.

$$\text{Drug release }\left(\%\right)=\frac{\text{The}\;\text{amount}\;\text{of}\;\text{Released}\;5-\text{Fu}\;}{\text{Amount}\;\text{of}\;\text{loaded}\;5-\text{Fu}}\times 100$$ (2)

### 2.8 *In-vitro* cytotoxicity

The cultivation of HCT-116 colorectal cancer cell lines had been started using RPMI-1640 media supplemented with 50 μg/mL of gentamycin alongside 10% of fetal calf serum. The cells were incubated at a temperature of 37°C and a CO_2_ concentration of 5%. The carcinogenic cell lines, with a density of 5 × 10^4^ cells per well, were cultivated for a duration of 3 weeks prior to being submerged in Corning^®^ 96-well plates over a period of 24 h. Next, specific doses of the 5-Fu-loaded ZP/HP, CS@ZP/HAP, and CD@ZP/HAP were introduced onto the cell strains, which were then subsequently cultivated over an additional 24 h. The quantification of viable cells produced during the time frame of incubation has been evaluated employing the commonly used MTT cell growth assay. The inserted medium for cultivation was effectively eliminated after completing this incubation phase and substituted with freshly formed medium (100 µL of RPMI). The recently introduced medium was well blended with the MTT (10 μL; 12 mM), and the mixture was further incubated over an additional 5 h to monitor the growth of formazan that is exhibiting a distinct purple coloration. The formazan that was produced was effectively removed by adding 50 µL of a DMSO solvent. Throughout the final step, a microplate has been employed for determining the optical density (OD) of cell lines that have been created through the examination. The microplate is adjusted to a specific wavelength of 590 nm. The cell viability % was determined using the calculated values, as stated in Equation 3 (Sayed et al., 2022a).

$$\text{Cell viability }\left(\%\right)=\frac{\text{Mean}\;\text{OD}}{\text{Control}\;\text{OD}\;}\times 100$$ (3)

### 2.9 Statistical analysis

The reported data have been included in their mean values ±the established standard errors for these mean data (S.E.M.), assuming the n value equals 3. The significance and accuracy of the statistical tests for the outcomes depend on the pairing tests and analyses of variance (ANOVA), where *p levels are less than 0.05.

## 3 Results and discussion

### 3.1 Characterization of the carrier

#### 3.1.1 XRD analysis

**FIGURE 1:**
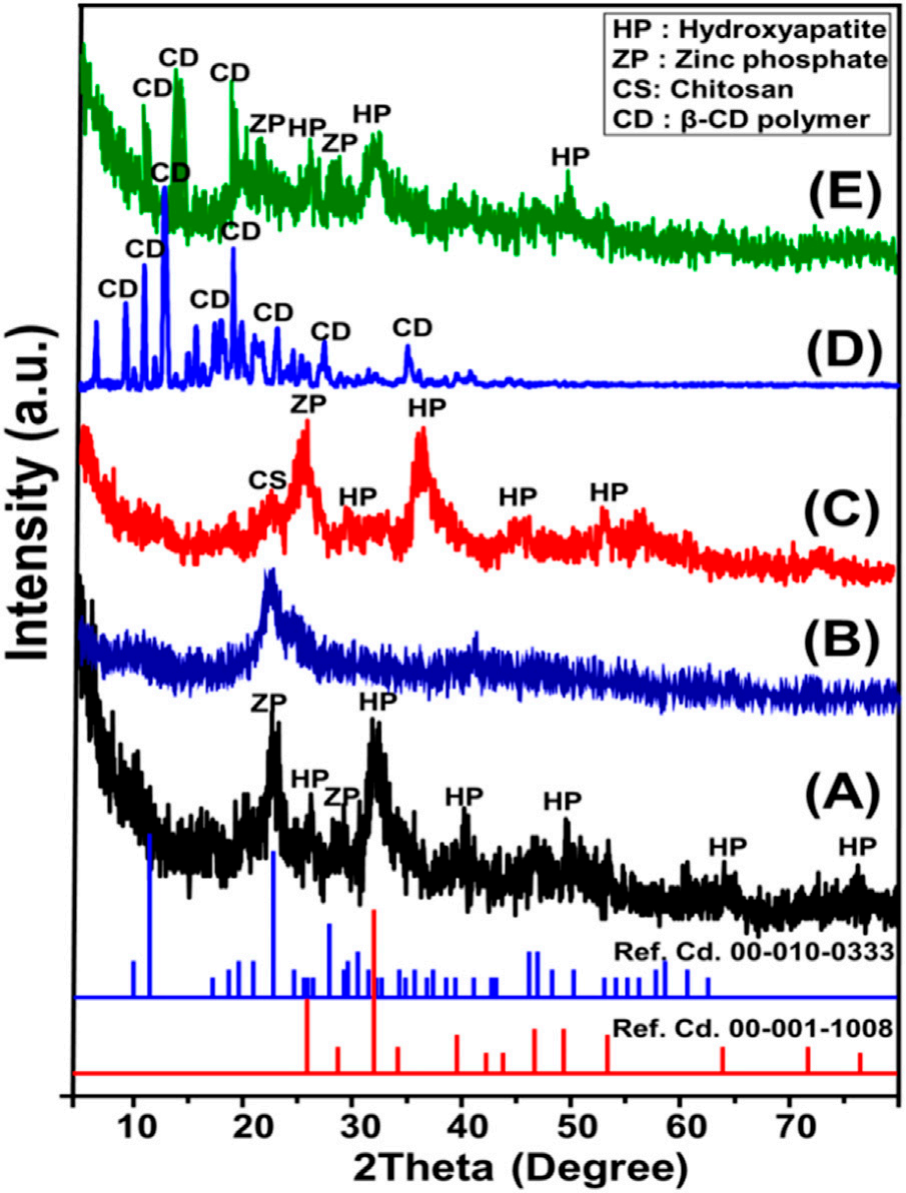
XRD patterns of ZP/HP **(A)**, CS polymer **(B)**, CS@ZP/HP composite **(C)**, β-CD **(D)**, and CD@ZP/HP composite **(E)**.

**FIGURE 2:**
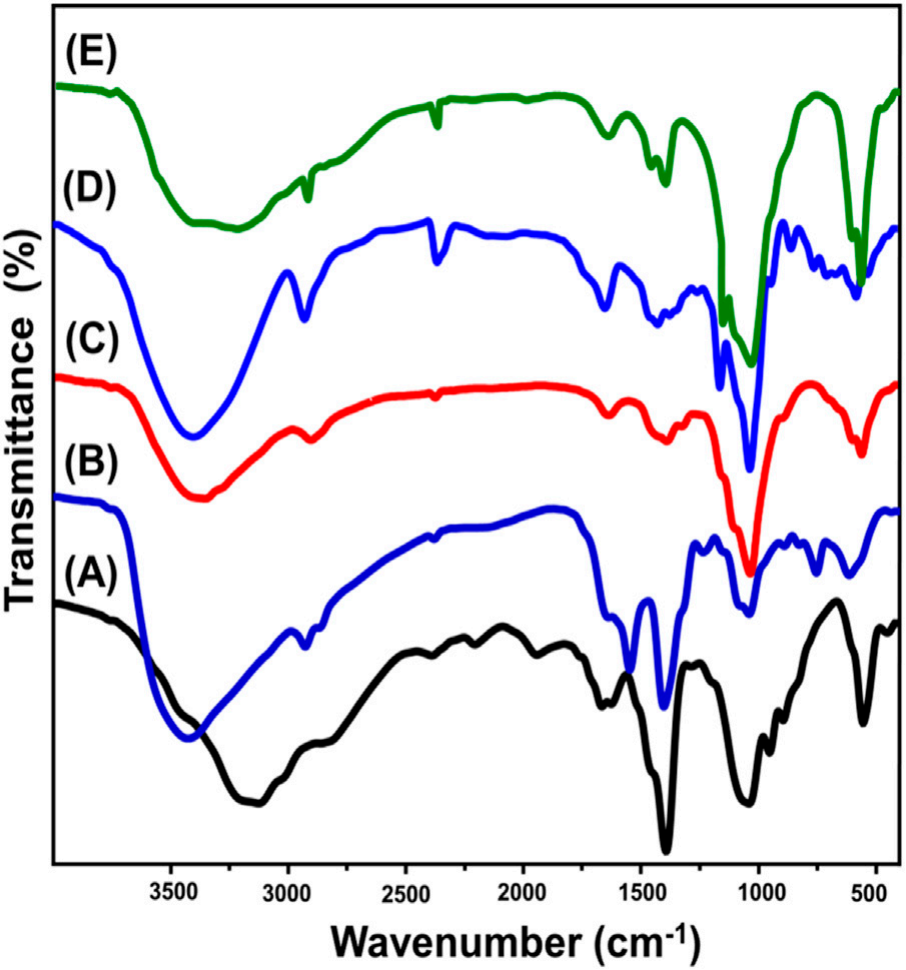
FT-IR spectra of ZP/HP **(A)**, CS polymer **(B)**, CS@ZP/HP composite **(C)**, β-CD **(D)**, and CD@ZP/HP composite **(E)**.

The X-ray diffraction patterns obtained for the ZP/HP, CS, CS@ZP/HP, β-CD, and CD@ZP/HP nanostructures have been presented in Figure 2. ZP/HAP discernible peaks reveal that the hydroxyapatite phase (HAP) (26.20°, 32.20°, and 39.60°) (Ref. Cd. 00-001-1008) effectively formed in a composite together with the zinc phosphate (ZP) phase (22.8°) (Ref. Cd. 00-010-0333) (Figure 1A). The HAP and ZP materials were successfully synthesized, with crystal dimensions of about 7.4 nm and 8.5 nm, respectively. The existence of a mixed structure consisting of two separate types of crystals could be attributed to the hypothesized substitution of the apatite exchangeable and structural ions as the first generated form by the extra Zn^2+^ ions inside the manufacturing solution (Sayed et al., 2022b; Chaudhry et al., 2022).

For the integrated CS, the signified peaks around 10° and 22° indicate that it comprises commercialized chitosan polymer, which exhibits semi-crystalline characteristics (Figure 1B). The CS@ZP/HP hybrid’s pattern exhibits the key peaks that characterize the ZP/HP structure; however, it displays a small shifting towards lower degrees (Figure 1C). Furthermore, one of the two major peaks related to chitosan had been eliminated, and the remaining peak was shifted and paired with the key peaks of HAP and ZP to generate an effective hybrid involving ZP/HP alongside chitosan. Regarding β-cyclodextrin as a separated ingredient, the marked pattern demonstrates its well-crystallized nature as a precursor used. It exhibits many intense peaks at certain 2 Theta angles, spanning from 6.80° until 34.90° (Figure 1D). The dominant peaks of ZP/HP were clearly discernible in the pattern of the CD@ZP/HP hybrid, in addition to some marked peaks of CD. Nevertheless, these peaks were somewhat shifted in the direction of lower angles, suggesting an effective integration between ZP/HP and β-CD (Figure 1E). This likely entails the formation of hydrogen-bonds and chemical complexes, along with the predicted insertion of β-CD inside the basic structure of ZP/HP (Figure 1E).

#### 3.1.2 FT-IR analysis

The integration and functional processes of ZP/HP, CS, CS@ZP/HP, β-CD, and CD@ZP/HP were evaluated depending on the corresponding FT-IR spectra (Figure 2). The spectrum of ZP/HP shows the main group existing within the HAP and ZP structures, which involves PO_4_^3−^ (at approximately 1,041 and 556 cm^−1^), Zn-O (at around 454 cm^−1^), HPO_4_^2−^ (at approximately 954 and 896 cm^−1^), and H-OH (at approximately 1,666 and 1,627 cm^−1^), as well as O-H (at approximately 3,125 cm^−1^) (Figure 2A) (Tao et al., 2021; Nguyen et al., 2020; Nagaraju et al., 2017; Wang, 2013). Additionally, other noticeable bands were identified correlated with N-H (NH_3_^−^) at about 1,390 cm^−1^ and CO_3_^2−^ with the spectrum range from 1943 to 2,376 cm^−1^) which signifies the chemical impact of the applied acids during the synthesis and CO_2_ gas from the atmosphere (Figure 2A) (Amiri et al., 2019; Mehta et al., 2018). When comparing the CS@ZP/HP spectrum (Figure 2C) with those corresponding to CS (Figure 2B), substantial variations can be noticed with respect to the spectral positions and magnitudes of the bands. Additionally, intricate bands corresponding to the two integrated types of CS [C-H (2,906 cm^−1^), C-N (1,404 cm^−1^), C=O (1,639 cm^−1^), and C-O (1,061 cm^−1^)] together with ZP/HP [PO_4_^3−^ (N-H group (1,392 cm^−1^), 1,037 and 564 cm^−1^), and CO_3_^2−^ group (2,377 cm^−1^)] are detected (Figure 2C) (Saad et al., 2020; Ibrahim et al., 2021). The observed variations in the locations and magnitudes of the various FT-IR bands, alongside the diminution of some IR bands that belong to important groups like HPO_4_^2−^ for ZP/HP and N-H of CS, verify the successful bonding among these active groups throughout the development of the blend. Comparing CD@ZP/HP (Figure 2E) to pure β-CD (Figure 2D), it has been noticed that its spectrum demonstrates the existence of different bands that correspond to the constituents of ZP/HP [OH (3,766 cm^−1^) and P-O (1,034 and 566 cm^−1^)] in addition to the comparable bands for β-CD (C=H (2,916 cm^−1^), hyperfine C=H (1,460 cm^−1^), and C=C (1,643 cm^−1^) (Figure 2E) (Krawczyk et al., 2022; Jiang et al., 2020). These complicated HAP and β-CD interconnected groups, together with significant changes in the approximate positions for distinguishing bands, have strengthened the effective formation of the CD@ZP/HAP composite.

#### 3.1.3 SEM and HRTE analyses

**FIGURE 3:**
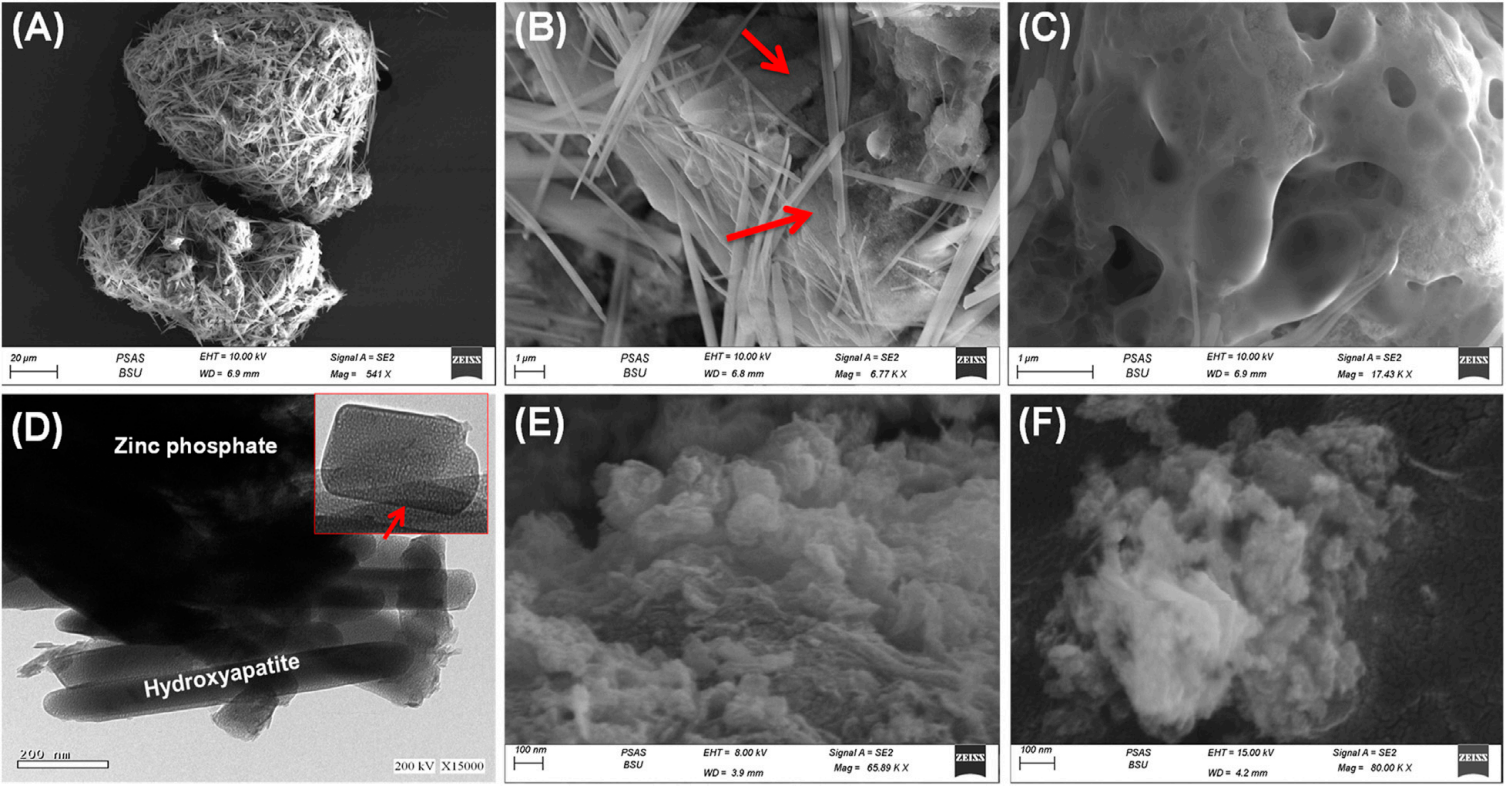
SEM images of synthetic ZP/HP **(A,B)**, high magnification SEM image on the ZP porous substrate **(C)**, HRTEM image of ZP/HP **(D)**, SEM image of synthesized CS@ZP/HP **(E)**, and SEM image of synthesized CD@ZP/HP composite **(F)**.

The SEM images readily demonstrated the heterogeneous composition of the ZP/HP hybrid and verified the existence of two distinct components, HP and ZP, which form a core-shell shape (Figures 3A,B). The ZP phase has been detected as aggregates of nanoparticles with massive geometric characteristics and extensively porous structures (Figure 3C). The observed pores have been classified into two distinct types: structurally developed and secondary types corresponding to micro-vugs, which have been generated as a result of the release of gaseous gas during the production steps (Figure 3C). The ZP base materials are heavily coated with HAP particles, which are easily identifiable as individual nano-rods exhibiting a clearly described shape and geometry. The observed rods possessed average widths that varied from 5 to 400 nm and an average length spanning from 1 to 10 µm. A notable nano-porous matrix was formed by the randomized crossings between these rods. The HRTEM images confirm the observations established through the SEM images, demonstrating the existence of HAP nano-rods together with spongy ZP as a basis (Figure 3D). Furthermore, the HRTEM image strongly revealed the very porous construction of the HAP nano-rods.

The SEM photographs of the synthesized particulates of the CS@ZP/HP hybrid reveal a notable blending of both the ZP/HP particulates and CS framework, with considerable realignment and rearranging of the HAP (Figure 3E). This provided a rough exterior and varied shaped blended grains, which included sphere-like, crooked, and wormy morphologies. The SEM images of the developed nanostructures of the CD@ZP/HP composite prove a significant integration between ZP/HP and the β-CD dense frameworks (Figure 3F). This integration leads to a noticeable alteration in the geometry of the resultant structure. Consequently, the exterior appeared uneven, and the combination of the granules displayed various shapes and orientations of the particulates that were merged. The HAP rods seemed to be bound together by the matrix of polymer, producing porous structure formed by the intersected rods (Figure 3F). The changes in geometry are associated with major changes in the textural properties, such as the surface area and the distribution of pore sizes. The ZP/HAP produced possesses a surface area of 138.2 m^2^/g. However, the synthesis of CS@ZP/HP and CD@ZP/HP leads to a little rise in the surface area, reaching 139.4 and 144.3 m^2^/g, respectively.

### 3.2 Encapsulation of 5-Fu

#### 3.2.1 Influence of the parameters

##### 3.2.1.1 Effect of pH

**FIGURE 4:**
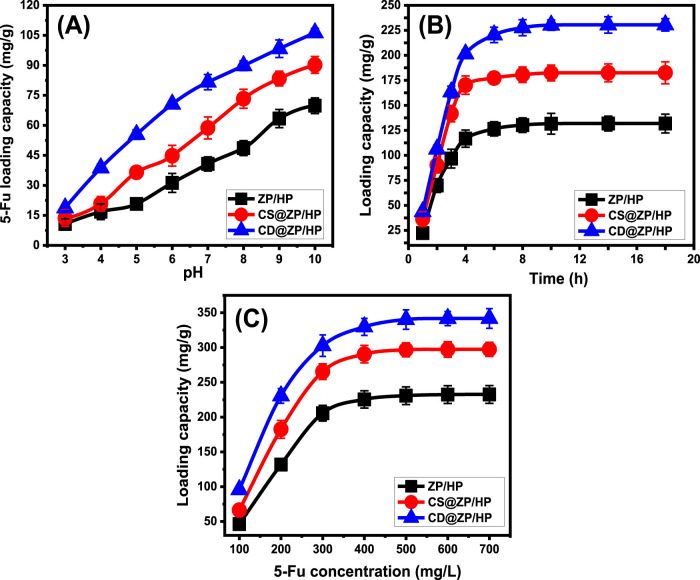
Effect of the experimental variables on the loading effectiveness of 5-Fu including **(A)** pH, **(B)** duration, and **(C)** starting concentration of 5-Fu.

The influence of the pH of the solutions being studied on the binding qualities of ZP/HP, CS@ZP/HP, and CD@ZP/HP was examined throughout a range from pH 3 to 10. This was completed after maintaining the additional parameters that might influence the outcomes [quantity: 20 mg; duration: 2 h; 5-Fu content: 200 mg/L; temperature: 30°C; volume: 50 mL]. The efficacy of encapsulating 5-Fu within ZP/HP, CS@ZP/HP, and CD@ZP/HP was greatly enhanced under elevated pH situations. This has been detected from pH 3 [ZP/HP (10.7 mg/g), CS@ZP/HP (13.6 mg/g), and CD@ZP/HP (18.7 mg/g)] until pH 10 [ZP/HP (69.8 mg/g), CS@ZP/HP (90.2 mg/g), and CD@ZP/HP (106.2 mg/g)] (Figure 4A). Consequently, it was recommended to encapsulate 5-Fu inside ZP/HP, CS@ZP/HP, and CD@ZP/HP by employing the reactions at alkaline pH settings. The pH level of the solutions being used has a significant impact on the ionizing behavior of 5-Fu, along with the dominating surface charges throughout ZP/HP, CS@ZP/HP, and CD@ZP/HP. The molecular composition of the 5-Fu medications has significant ionizing activities beneath alkaline settings, as opposed to its activities beneath acidic to neutral settings (Tian et al., 2020; Sayed et al., 2022a). The increase in ionizing levels greatly affects the mobility, diffusion, and collisions of the 5-Fu ions with the reactive and empty loading sites, thereby enhancing the loading rates within the alkaline conditions.

##### 3.2.1.2 Loading duration

Experiments have been conducted over a period of 1–18 h to investigate the effects of durations on ZP/HP, CS@ZP/HP, and CD@ZP/HP loading effectiveness while keeping the rest of the parameters constant (pH: 10, volume: 50 mL, 5-Fu extent: 200 mg/L, temperature: 30°C, quantity: 20 mg). During the investigation, loading rates and quantities in mg/g demonstrated a clear improvement using ZP/HP, CS@ZP/HP, and CD@ZP/HP (Figure 4B). The improvement is evident within a time frame of 1–4 h for ZP/HP as well as CS@ZP/HP and last up to 10 h for CD@ZP/HP. Following these time frames, extending the duration of assessment had a negligible influence either on the speed of loading or the total amount of 5-Fu loaded. Over these periods, the diagrams show comparable results exhibiting consistent tendencies (Figure 4B). These properties reveal the equilibrium conditions of ZP/HP, CS@ZP/HP, and CD@ZP/HP, alongside the respective equilibrium abilities [131.8 mg/g (ZP/HP), 182.5 mg/g (CS@ZP/HP), and 230.5 mg/g (CD@ZP/HP)] (Figure 4B). The high levels of loading and sharp increase in the 5-Fu trapped quantities tracked throughout the initial phases of the encapsulating operations may be explained by the presence of functional binding receptors in their free forms and numerous numbers through the interfaces of ZP/HP, CS@ZP/HP, and CD@ZP/HP (Abukhadra et al., 2022). The number and availability of vacant sites within ZP/HP, CS@ZP/HP, and CD@ZP/HP have significantly dropped due to the ongoing occupancy by 5-Fu during the tests. Consequently, over time, the quantifiable rate at which 5-Fu had been bound decreased significantly by the end of the examination duration. Furthermore, there were no significant enhancements observed in the loading properties. Upon attaining saturation, the sites were completely occupied by 5-Fu molecules, resulting in the establishment of loading equilibrium states for ZP/HP, CS@ZP/HP, and CD@ZP/HP (Salam et al., 2022).

##### 3.2.1.3 5-Fu concentration

Different concentrations of 5-Fu have been analyzed to assess their influence on the loading characteristics of ZP/HP, CS@ZP/HP, and CD@ZP/HP. The concentration of 5-Fu varied between 100 and 700 mg/L, and other factors such as time (20 h), dose (20 mg), temperature (30°C), pH (9), and volume (50 mL) were kept constant. The best possible efficiency as well as equilibrium characteristics of ZP/HP, CS@ZP/HP, and CD@ZP/HP essentially depend on the starting levels of 5-Fu. At higher initial concentrations of 5-Fu, there was a significant rise in the total quantity of 5-Fu loaded into ZP/HP, CS@ZP/HP, and CD@ZP/HP. The driving forces and migrating qualities of these 5-Fu ions are significantly amplified due to their high concentration inside a specific volume. This increases the chances of interactions and promotes the chemical binding of the medication molecules to the existing receptors of ZP/HP, CS@ZP/HP, and CD@ZP/HP (Jiang et al., 2020; Salam et al., 2020). As a result, the loading effectiveness of 5-Fu into ZP/HP, CS@ZP/HP, and CD@ZP/HP has been strengthened up to particular concentrations (400 mg/L for ZP/HP and CS@ZP/HP and 500 mg/L for CD@ZP/HP). Any additional rise in the assessed 5-Fu contents after the prior threshold levels possesses no effect on the measurable loaded quantities of the drug, which generally correlate with the respective equilibrium states of ZP/HP, CS@ZP/HP, and CD@ZP/HP (Figure 4C). Thus, ZP/HP, CS@ZP/HP, and CD@ZP/HP achieve their maximum loading potentials, which are 232.6 mg/g, 297.4 mg/g, and 341.8 mg/g, respectively. The improved encapsulation efficiency of CS@ZP/HP and CD@ZP/HP compared to ZP/HP may be attributed to several aspects. These include the expanded surface area after modifications, the organophilic properties of CS@ZP/HP and CD@ZP/HP, which improve their affinities for 5-Fu, along with the additional binding sites resulting from the integration process.

#### 3.2.2 Loading mechanism

##### 3.2.2.1 Kinetic properties

###### 3.2.2.1.1 Intra-particle diffusion behavior

**FIGURE 5:**
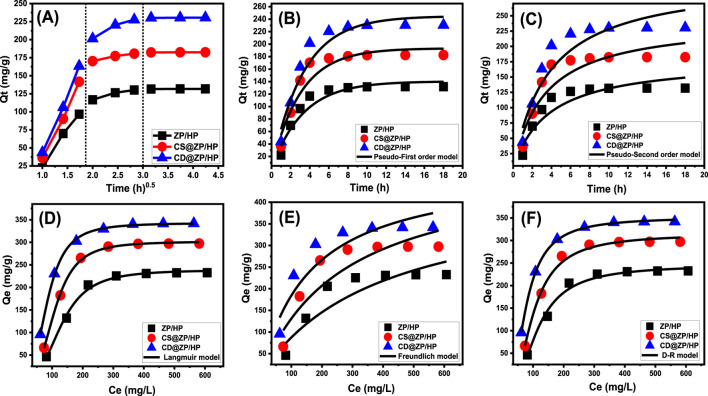
Intra-particle diffusion curves of the loading activities of 5-Fu **(A)**, fitting of the loading activities with kinetic models **(B,C)**, and fitting of the loading results with the classic isotherm models **(D–F)**.

The intra-particle diffusion patterns of the encapsulation activities of 5-Fu into ZP/HP, CS@ZP/HP, and CD@ZP/HP (Figure 5A) could be divided into three different phases, with no crosses with the starting positions of the curves. This emphasizes that there are collaborative processes underlying the 5-Fu loading alongside the key function represented by the medication ions’ diffusing pathways towards the ZP/HP, CS@ZP/HP, and CD@ZP/HP interaction surfaces (Salam et al., 2020; El Qada, 2020). This may include the process of molecules bonding to the functional binding sites of the outer surface, layered loading reactions, ion diffusion effects, and the impact of attaining a state of saturation or equilibrium (Lin et al., 2021). The first segment refers to the occurrence of external loading reactions throughout the beginning phases of the examination (Figure 5A). The ongoing development of the encapsulation reactions is determined by the quantity of receptors that exist on the surfaces of ZP/HP, CS@ZP/HP, and CD@ZP/HP (Albukhari et al., 2021). An extra stage was observed by extending the time (Figure 5A). This stage demonstrates the participation of many regulatory mechanisms, including the impact of layered loading activities by internal receptors alongside the diffusion characteristics of 5-Fu. The third stage was identified as a key part corresponding to the equilibrium settings for 5-Fu encapsulation into ZP/HP, CS@ZP/HP, and CD@ZP/HP. This validates the occupancy or utilization of all the operational receptors with 5-Fu ions (Figure 5A) (Sayed et al., 2022a; Salam et al., 2020). The binding behaviors occurring during this phase are controlled by multiple processes that could involve molecular and/or interionic attractions (Jiang et al., 2020).

###### 3.2.2.1.2 Kinetic modeling

The kinetic characteristics of the activities involved in embedding 5-Fu within ZP/HP, CS@ZP/HP, and CD@ZP/HP have been analyzed based on the theoretical principles of the pseudo-first-order (P.F.) (Equation 4) and pseudo-second-order (P.S.) (Equation 5) hypotheses. The agreement between the 5-Fu binding behaviors and the kinetic concepts of both of these models was evaluated by non-linear fits utilizing their descriptive formulas. The correlation coefficient (R^2^) and Chi-squared (χ^2^) have been employed as metrics for the model’s efficacy in matching the results.

$$Q_{t\;}=Q_{e}\;\left(1-e^{{-k}_{1}.t}\right)$$ (4)

$$Q_{t}=\frac{Q_{e\;}^{2}k_{2}t}{1+Q_{e}k_{2}t}$$ (5)

The obtained R^2^ and χ^2^ values reveal that the kinetic properties of the P.F. concept (Figure 5B; Table 1) provide a more accurate description of the 5-Fu encapsulation behaviors inside ZP/HP, CS@ZP/HP, and CD@ZP/HP in comparison with the P.S. theory (Figure 5C; Table 1). The experimentally measured equilibrium capacities [131.8 mg/g (ZP/HP), 182.5 mg/g (CS@ZP/HP), and 230.5 mg/g (CD@ZP/HP)] were highly consistent with the expected levels obtained from the P.F. modeling (140.3 mg/g for ZP/HP, 193.3 mg/g for CS@ZP/HP, and 244.9 mg/g for CD@ZP/HP), thus validating the accuracy of the fitting results (Table 1). The entrapment of 5-Fu within ZP/HP, CS@ZP/HP, and CD@ZP/HP mostly occurred due to physical processes, including van der Waals forces and electrostatic forces. The specific mechanism depended on the kinetic characteristics of the P.F. model (Chaima et al., 2024; Sherlala et al., 2019). However, the P.F. concept provides a more precise illustration for the loading processes compared to the P.S. concept. Despite this, the fitting findings still align significantly with those of the P.S. concept. Poor chemical mechanisms such as hydrogen bonding, electron sharing, and hydrophobic bonds were assumed to have a minimal impact or offer little support during the loading of 5-Fu within ZP/HP, CS@ZP/HP, and CD@ZP/HP (Salam et al., 2020; Sherlala et al., 2019). The combination of both chemical and physical interactions led to the establishment of a drug-loaded layer by means of chemical events, which served as a foundation for the subsequent establishment of a further drug-loaded layer encompassing physical processes (Jasper et al., 2020).

**TABLE 1:** The determined theoretical variables for the loading kinetics, equilibrium, and release kinetic models under study.

Model	Parameters	ZP/HP	CS@ZP/HP	CD@ZP/HP
Kinetic models				
Pseudo-first-order	K_1_ (min^−1^)	0.304	0.328	0.300
	Qe _(Cal)_ (mg/g)	140.3	193.3	244.9
	R^2^	0.93	0.94	0.95
	X^2^	2.26	2.64	2.62
Pseudo-second-order	k_2_ (g mg^-1^ min^-1^)	0.00152	0.00125	8.69 × 10^−4^
	Qe _(Cal)_ (mg/g)	179.4	243.06	312.3
	R^2^	0.89	0.89	0.91
	X^2^	3.53	4.4	4.58
Isotherm models				
Langmuir	Q_max (mg/g)_	238.9	301.7	342.8
	b (L/mg)	3.74 × 10^−7^	2.78 × 10^−7^	2.40 × 10^−6^
	R^2^	0.99	0.99	0.99
	X^2^	0.259	0.060	0.033
	RL	0.99	0.98	0.98
Freundlich	1/n	0.642	0.568	0.457
	k_F_ (mg/g)	4.52	9.4	21.7
	R^2^	0.85	0.83	0.87
	X^2^	8.2	8.6	7.08
D-R model	β (mol^2^/KJ^2^)	0.0110	0.00801	0.00774
	Q_m_ (mg/g)	245.9	314.4	351.4
	R^2^	0.99	0.99	0.99
	X^2^	0.51	0.28	0.20
	E (KJ/mol)	6.74	7.9	8.03
Monolayer model of one energy	n	3.03	3.21	2.91
	Nm (mg/g)	78.85	93.87	117.8
	Q_(sat)_ (mg/g)	238.9	301.3	342.8
	∆E (kJ/mol)	−5.76	−4.68	−4.12

Release kinetics						
	Determination coefficient					
	ZP/HP		CS@ZP/HP		CD@ZP/HP	
	pH 1.2	pH 7.4	pH 1.2	pH 7.4	pH 1.2	pH 7.4
Zero-order	0.75	0.71	0.63	0.49	0.59	0.47
First order	0.98	0.89	0.94	0.98	0.94	0.97
Higuchi	0.93	0.89	0.82	0.75	0.80	0.70
Hixson-Crowell	0.92	0.83	0.86	0.95	0.85	0.97
Korsmeyer-peppas	0.86	0.85	0.86	0.82	0.86	0.83
n	0.67	0.72	0.59	0.51	0.57	0.48

##### 3.2.2.2 Isotherm properties

###### 3.2.2.2.1 Classic isotherm models

Applying the Langmuir (Equation 6) and Freundlich (Equation 7) mathematical models alongside Dubinin-Radushkevich (D-R) (Equation 8) parameters, the equilibrium aspects of incorporating 5-Fu into ZP/HP, CS@ZP/HP, and CD@ZP/HP as possible carriers have been investigated. The measurements were fitted utilizing the specified equations of each model through a non-linear approach. The degree of agreement was determined based on the correlation coefficient (R^2^) and chi-squared (χ^2^) (Table 1; Figures 5D–F).

$$Q_{e}=\frac{Q_{\max}\;bC_{e}}{\left(1+bC_{e}\right)}$$ (6)

$$Q_{e}=K_{f}C_{e}^{1/n}$$ (7)

$$Q_{e}=Q_{m}e^{-\beta {ɛ}^{2}}$$ (8)

The insertion of 5-Fu into ZP/HP, CS@ZP/HP, and CD@ZP/HP follows the Langmuir isotherm characteristics rather than the suggested Freundlich theoretical terms based on the provided model-fitting factors. Consequently, 5-Fu ions had been homogeneously retained across the surfaces of ZP/HP, CS@ZP/HP, and CD@ZP/HP, developing a monolayer of adsorbed 5-Fu via the existence of frequently and uniformly distributed reacting sites (Sayed et al., 2022a; Albukhari et al., 2021). The preferential encapsulation of 5-Fu molecules inside ZP/HP, CS@ZP/HP, and CD@ZP/HP carriers could be distinguished based on the calculated RL parameters with values that are less than 1.0. The maximum estimated loading capacities of 5-Fu in ZP/HP, CS@ZP/HP, and CD@ZP/HP have been established employing the Langmuir isotherm criteria and determined to be 238.9 mg/g, 301.7 mg/g, and 342.8 mg/g, respectively (Table 1).

The equilibrium properties of the examined D-R model could reveal valuable information on the energy fluctuations throughout the exteriors of ZP/HP, CS@ZP/HP, and CD@ZP/HP as carriers of 5-Fu, regardless of their degree of homogeneity or heterogeneity (Radjai et al., 2022). The Gaussian energy (E) can easily be determined using D-R modeling, which is useful during the identification of the principal loading techniques if they are regulated by chemical or physical interactions. The physical processes have a Gaussian energy distribution under 8 kJ/mol, whereas the chemical processes exhibit values that exceed 16 kJ/mol. The existence of complex systems or poor chemical reactions may be inferred from Gaussian energies that vary between 8 and 16 kJ/mol (Sayed et al., 2022a; Radjai et al., 2022). The Gaussian energy during the entrapment of 5-Fu inside ZP/HP, CS@ZP/HP, and CD@ZP/HP were 6.74 kJ/mol, 7.9 kJ/mol, and 8.03 kJ/mol, respectively (Table 1). The results revealed that the physical processes play a substantial role during the capture of 5-Fu via ZP/HP, and there is also a possible impact of ion exchange processes that possess energy values varying from 0.6 kJ/mol until 25 kJ/mol. Nevertheless, the capture of these molecules via CS@ZP/HP and CD@ZP/HP matches the established range of collaborative physical and weak chemical mechanisms that include ion exchange events.

###### 3.2.2.2.2 Advanced isotherm models

**FIGURE 6:**
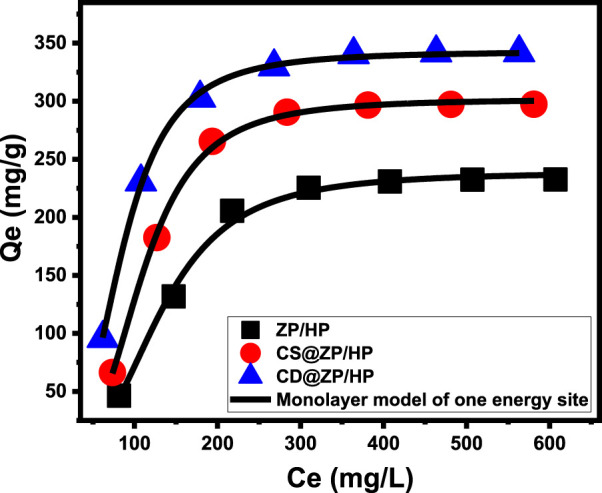
Fitting of the loading results with the advanced Monolayer model of one energy site.

The described enhanced isotherm models, based on the fundamental concepts of statistical physics theory, donate a deeper knowledge about the loading mechanisms in terms of the 5-Fu/carrier interfaces. The study implemented a monolayer model involving a single energy site (Equation 9) alongside several mathematical parameters, which include the steric and energetic aspects. The aforementioned approach facilitated the examination of the loading characteristics and the basic regulated mechanistic activities. The main factors influencing the fitting degrees included the determination coefficients (R^2^) and the root mean square error (RMSE) (Figure 6; Table 1).

$$Q=nN_{o}=\frac{nN_{M}}{1+{\left(\frac{C1/2}{\text{Ce}}\right)}^{n}}=\frac{Q_{o}}{1+{\left(\frac{C1/2}{\text{Ce}}\right)}^{n}}$$ (9)

The statistical analysis of the model’s parameters revealed several steric factors, including the average density of available and functional loading binding sites [Nm_(5-Fu)_] across ZP/HP, CS@ZP/HP, and CD@ZP/HP. It additionally assessed the number of 5-Fu ions captured within just one operational receptor [n_(5-Fu)_] and the corresponding saturation loading quantities [Qsat_(5-Fu)_]. The determined energetic aspect corresponds to the energy of the 5-Fu loading (E). The overall density of the successful loading sites increased significantly after alterations of ZP/HP to produce CS@ZP/HP (93.8 mg/g) and CD@ZP/HP (117.8 mg/g), relative to the beginning value of Nm_(5-Fu)_ = 78.8 mg/g (Table 1). This could potentially be due to the incorporation of more active and free functional groups, which correlate with the combination of chitosan and β-CD, or it might occur as a result of the improvement in the surface area that arises from the interactions. After these modifications, the saturating values had a significant rise from 238.9 mg/g (ZP/HP) to 301.3 mg/g (CS@ZP/HP) and 342.8 mg/g (CD@ZP/HP), respectively (Table 1). The number of molecules of 5-Fu loaded onto every single active site across ZP/HP, CS@ZP/HP, and CD@ZP/HP (n _(5-Fu)_) indicates the considerable influence of the modification processes on the characteristics of the ZP/HP surface as a drug carrier, especially via β-CD. According to mathematical computations, the quantity of 5-Fu ions bound to each individual site throughout ZP/HP, CS@ZP/HP, and CD@ZP/HP is 303, 3.21, and 2.9, respectively (Table 1). These numbers exceed 1, which signifies the vertical arrangement of the filled ions over the exterior interfaces and their encapsulation by multi-molecular mechanisms (Yang et al., 2022; Sellaoui et al., 2016).

The loading energies (E) were computed using Equation 10 (Table 1), implementing the theoretically estimated residual 5-Fu concentrations at the half saturation point (C1/2) along with their solubility.

$$∆E=-RT\;\ln\left(\frac{S}{C_{1/2}}\right)$$ (10)

The binding energies computed for loading 5-Fu into ZP/HP, CS@ZP/HP, and CD@ZP/HP were −5.7 kJ/mol, −4.6 kJ/mol, and −4.11 kJ/mol, respectively (Table 1). The results validate the most recent discoveries on the physical encapsulation mechanisms of 5-Fu into ZP/HP, CS@ZP/HP, and CD@ZP/HP (Yang et al., 2022). The mechanisms involved in this process comprise van der Waals forces, which exhibit an energy level ranging from 4 to 10 kJ/mol, dipole forces exhibiting an energy level ranging from 2 to 29 kJ/mol, and hydrogen bonding exhibiting an energy level below 30 kJ/mol (Ashraf et al., 2022; Ali et al., 2021).

### 3.3 *In-vitro* release profiles

**FIGURE 7:**
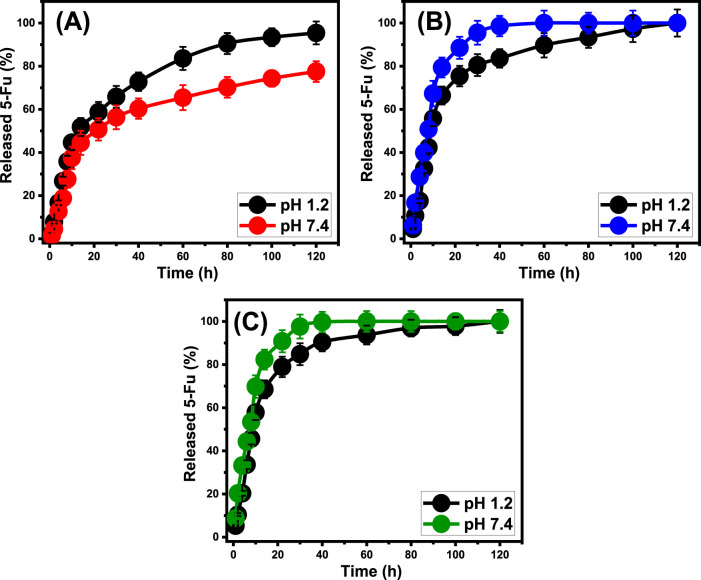
*In-vitro* release profiles of **(A)** ZP/HP, **(B)** CS@ZP/HP, and **(C)** CD@ZP/HP as carriers of 5-Fu.

The release behaviors of ZP/HP, CS@ZP/HP, and CD@ZP/HP have been evaluated by quantifying the quantities of 5-Fu molecules that diffused into gastric fluid (pH 1.2) as well as intestinal fluid (pH 7.4), which have been employed as analogues to mimic the environment of malignant cells (Figure 7). The measured diffusing% of 5-Fu out of ZP/HP, CS@ZP/HP, and CD@ZP/HP within the two tested buffers demonstrate significant differences in the detected rates corresponding to the rise in the releasing periods (Figures 7A–C). The diffusion rates of 5-Fu out of ZP/HP, CS@ZP/HP, and CD@ZP/HP exhibit rapid features that are correlated with substantial variations in the amounts of 5-Fu liberated. Following particular release intervals, the measurable rates of diffusing of 5-Fu reduced dramatically, whereas there was no noticeable enhancement in the amounts that diffused (Figure 7). At this point, the releasing behaviors of ZP/HP, CS@ZP/HP, and CD@ZP/HP had attained a stable state. The rapid diffusion behaviors of 5-Fu noticed throughout the starting release intervals have been ascribed to the abrupt releasing of loosely bonded along with physically filled 5-Fu ions from the exterior loading sites throughout ZP/HP, CS@ZP/HP, and CD@ZP/HP (AbouAitah et al., 2021; Wang et al., 2021; Mostafa et al., 2021). After the entirely desorption process of these slightly embedded and surficial trapped 5-Fu ions, their liberation features were regulated by chemically embedded ions in addition to the encapsulated 5-Fu ions within the structurally constructed pores of ZP/HP, CS@ZP/HP, and CD@ZP/HP, which adversely affected the determined diffusion rates (Figure 7) (Tian et al., 2020; Elboraey et al., 2021; Sun et al., 2017). The high ionizing and dissolving qualities of 5-Fu under alkaline settings enhance the diffusion qualities of its ions out of CS@ZP/HP and CD@ZP/HP in the intestinal fluid in comparison with pH 1.2 (gastric fluid) (Vatanparast and Shariatinia, 2018; Rehman et al., 2018). Nevertheless, the ionizing characteristics of 5-Fu cause it to be release and diffuse more rapidly in basic situations, ZP/HP exhibits quicker characteristics at pH 1.2 compared to pH 7.4. The behavior that has been identified may be attributed to the poor stability and considerable breakdown characteristics of the HAP framework under acidic situations, leading to rapid diffusion of 5-Fu from ZP/HP at pH 1.2.

The release behaviors of 5-Fu from ZP/HP in gastric and intestinal fluids were maintained for a duration of 120 h. After being exposed to a pH of 1.2 for 14 h and a pH of 7.4 for 22 h, almost 50% of the 5-Fu content was expelled out of the ZP/HP framework (Figure 7A). After 100 h, the release of 5-Fu in stomach and intestinal fluids was not confirmed to be complete, with 95.4% and 77.5%, respectively (Figure 7A). The expected significant hydrogen bonds that occur between the 5-Fu ions and the predominant reactive functional structures of HAP could be responsible for the observed slow release behavior of it (Rehman et al., 2018). This prevents the 5-Fu from being released out of the apatite framework, which restricts the medication’s potential to be delivered successfully at the curative dosage. The releasing patterns of 5-Fu from CS@ZP/HP exhibit quicker features compared to the patterns of ZP/HP, regardless of whether the pH is 1.2 or 7.4 (Figure 7B). Following exposure to pH 1.2 for 10 h and pH 7.4 for 8 h, about 50% of the initial quantity of 5-Fu diffused out of the CS@ZP/HP structure (Figure 7B). The complete release of 5-Fu into gastric and intestinal fluids has been observed after 120 h and 80 h, respectively (Figure 7B). The combination of ZP/HP with β-CD (CD@ZP/HP) similarly led to an elevation in the releasing properties of 5-Fu ions (Figure 7C). Following a 10-h exposure to a pH of 1.2 and a further 8-h exposure to a pH of 7.4, almost 50% of the 5-Fu compound was successfully liberated from the CD@ZP/HP framework. The entire release of 5-Fu in the intestinal and stomach fluids had been observed within 120 and 60 h, respectively (Figure 7C).

The rise in release rate following the formation of hybrids composed of ZP/HP together with chitosan and β-cyclodextrin can be attributed to their functions as coatings or barriers that exist between the functional groups of the HAP framework and the molecular structure of 5-Fu. It hindered the anticipated formation of hydrogen bonds between the drug’s framework and the hydroxyl-containing effective chemical units of ZP/HP, resulting in the fast diffusion of the 5-Fu molecules. Additionally, a consistent distribution of the mentioned 5-Fu ions inside the polymeric chains significantly enhances the rate at which the medication diffuses (Dziadkowiec et al., 2017). Furthermore, the inclusion of such polymers results in the integration of more reactive sites, hence is enhancing the possibility of the 5-Fu molecules that were physically bound across these exterior sites. For conditions that necessitate extended interactions and contact involving medicine ions and malignant cells, it is recommended to use the controlled diffusing of 5-Fu as an antitumor drug (Tian et al., 2020; Sundaramoorthy et al., 2016). Moreover, in certain settings where particular curative dosages necessity to be given rapidly, it is advisable to use sudden and accelerated administration systems. The synthesized CS@ZP/HP and CD@ZP/HP have the potential to operate as drivers of 5-Fu, providing a controlled delivery approach with managed encapsulation and releasing properties.

### 3.4 Release kinetic studies

Kinetic studies were conducted on the 5-Fu releasing behaviors from ZP/HP, CS@ZP/HP, and CD@ZP/HP as indicators of the suitably controlled mechanistic processes. The releasing processes were simulated employing zero-order (Z-O) (Equation 11), first-order (F-O) (Equation 12), Higuchi (H-G) (Equation 13), Hixson-Crowell (H-C) (Equation 14), and Korsmeyer-Peppas (K-P) (Equation 15) kinetic mathematical models. The modeling methods have been emphasized according to the degrees of linear regression matching (Tian et al., 2020).

$$W_{t}-W_{0}=K_{0}.t$$ (11)

$$\ln\left(\;W_{\infty }/W_{t}\right)=K_{1}.t$$ (12)

$$W_{t}=K_{h}t^{1/2}$$ (13)

$$W_{o}^{1/3}-W_{t}^{1/3}=K_{HC}t$$ (14)

$$W_{t}/\;W_{\infty }=K_{p\;}t^{n}$$ (15)

The zero-order kinetics indicate that the diffusing of 5-Fu via ZP/HP, CS@ZP/HP, and CD@ZP/HP occurs at constant rates, regardless of the amount of drug loaded (Othman et al., 2021). The effectiveness of releasing the 5-Fu-loaded doses from ZP/HP, CS@ZP/HP, and CD@ZP/HP is greatly influenced by the F-O hypothesis. The Higuchi kinetics hypothesis proposes that diffusion-related activities have the key effect during release events (El-Zeiny et al., 2020; Ge et al., 2019). The diffusion operations, which followed Higuchi kinetics, occurred at a constant rate that was smaller than the starting dosage of 5-Fu. Furthermore, the carriers implemented must exhibit desirable sink properties, and their swelling or dissolving must not have any effect on the release characteristics (Othman et al., 2021). The kinetic concepts of the Hixson-Crowell theory concentrate on erosion responses rather than diffusion processes. The surface area and grain size of the evaluated drivers have a substantial impact on their behaviors (Othman et al., 2021; Ibrahim et al., 2021). The Korsmeyer-Peppas kinetic mechanistic hypothesis indicates that the releasing activities comprise the combined impact of diffusing and erosion pathways working together (El-Hamshary et al., 2019).

**FIGURE 8:**
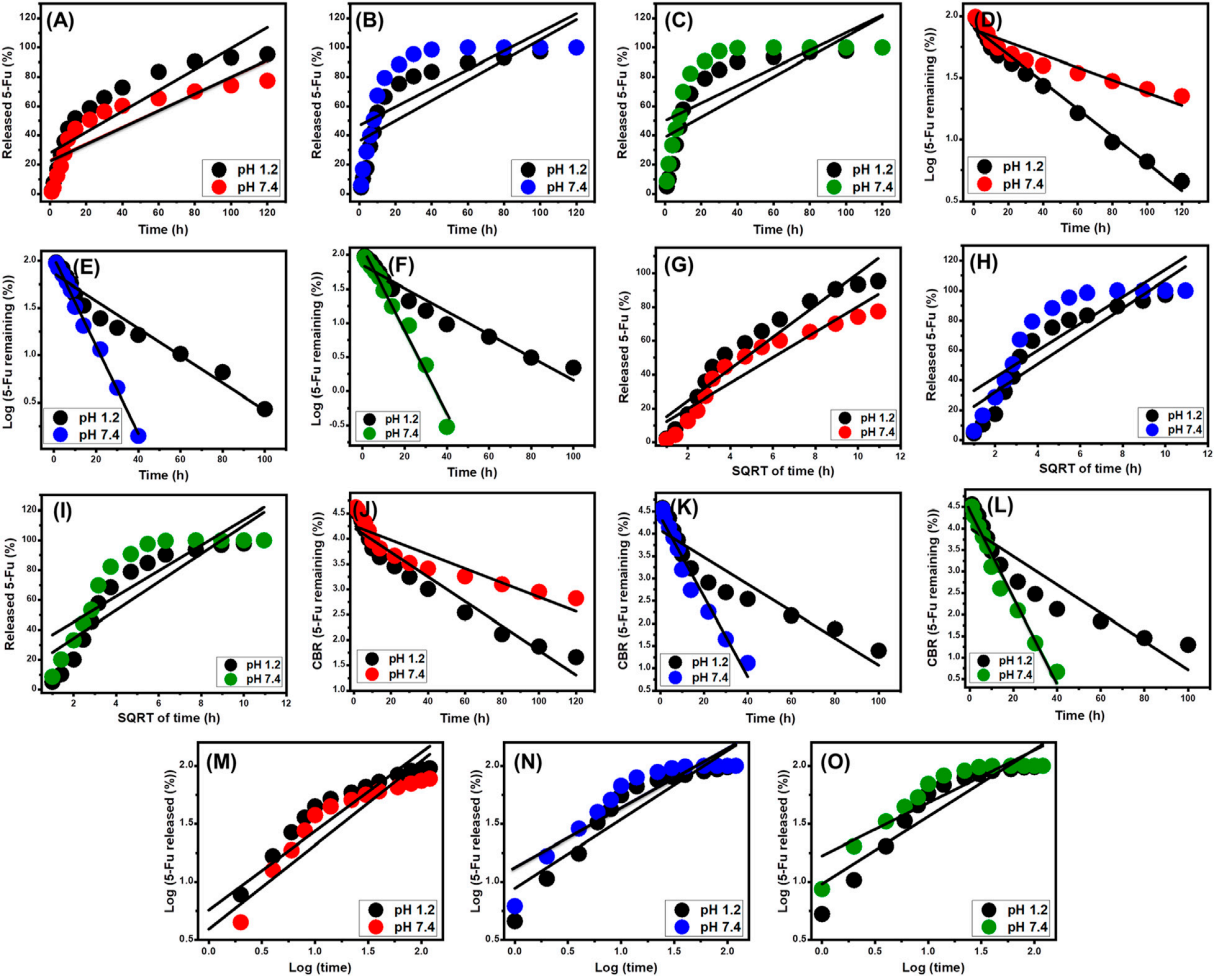
The linear regression fitting of the 5-Fu releasing results with Z-O [ZP/HP **(A)**, CS@ZP/HP **(B)**, and CD@ZP/HP **(C)**], F-O [ZP/HP **(D)**, CS@ZP/HP **(E)**, and CD@ZP/HP **(F)**], H-G [ZP/HP **(G)**, CS@ZP/HP **(H)**, and CD@ZP/HP **(I)**], H-C [(ZP/HP **(J)**, CS@ZP/HP **(K)**, and CD@ZP/HP **(L)**], and K-P [ZP/HP **(M)**, CS@ZP/HP **(N)**, and CD@ZP/HP. **(O)**] release kinetic models.

The R^2^ results indicate that the releasing processes of 5-Fu from ZP/HP, CS@ZP/HP, and CD@ZP/HP adhere to the F-O (Figures 8D–F) kinetic concept rather than the Z-O theory (Figures 8A–C). This suggests that the amount of preloaded 5-Fu has a substantial impact on the release effectiveness. The release properties align effectively with the two different Higuchi (H-G) (Figures 8G–I) and Hixson-Crowell (H-C) (Figures 8J–L) hypotheses. The kinetic evaluation findings confirmed the collaborative involvement of diffusion and erosion mechanisms in promoting the release of 5-Fu. However, the release patterns of CS@ZP/HP and CD@ZP/HP closely correspond to the Hixson-Crowell kinetics, indicating the significant influence of erosion processes. The close correlation noticed between the releasing characteristics of 5-Fu and the Korsmeyer-Peppas hypothesis (Figures 8M–O), in conjunction with the calculated diffusion exponent (n), provides further evidence for the complex mechanistic explanations. The diffusion exponent (n) values exceed 0.45, indicating that the release mechanisms of 5-Fu by the ZP/HP, CS@ZP/HP, and CD@ZP/HP drivers have non-Fickian transport features (El-Hamshary et al., 2019).

### 3.5 Cytotoxicity properties

**FIGURE 9:**
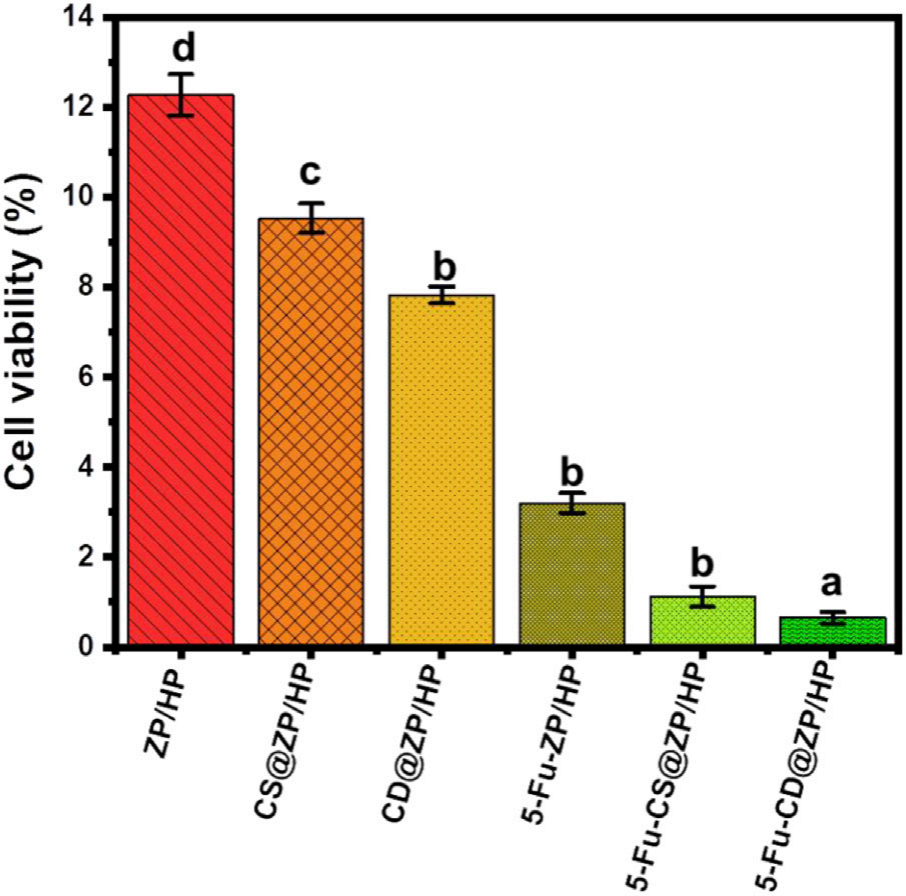
Cytotoxicity properties of ZP/HP, CS@ZP/HP, and CD@ZP/HP as well as their 5-Fu loaded forms. The values are the averages of five replicates, the error bars indicate the standard error of means, and different letters specify a statistical difference between the means (*p* < 0.05).

The cytotoxic effects of free ZP/HP, CS@ZP/HP, and CD@ZP/HP particles on HCT-116 tumor cells were investigated. The developed products, whenever used as unloaded particles, revealed significant toxicities against tumor cells, especially at dosages up to 500 μg/mL (Figure 9). At a concentration of 500 μg/mL, the cell viability % for the empty ZP/HP, CS@ZP/HP, and CD@ZP/HP particles have been estimated to be 12.28%, 9.53%, and 7.8%, respectively (Figure 9). The cytotoxicity findings confirm that the biological significance of ZP/HP is greatly augmented when combined with chitosan (CS@ZP/HP) and β-CD (CD@ZP/HP). Cell toxicity characteristics of 5-Fu-encapsulated ZP/HP, CS@ZP/HP, and CD@ZP/HP were evaluated by treating cell strains with a dose of 500 μg/mL. The cell viability % obtained were 3.2%, 1.12%, and 0.63%, respectively (Figure 9). The cytotoxic results demonstrate the strengthened effect of the ZP/HP, CS@ZP/HP, and CD@ZP/HP carriers upon the cytotoxic remarks and the curative effectiveness of the 5-Fu therapy.

## 4 Conclusion

The core-shell nanostructure of a zinc-phosphate/hydroxyapatite hybrid (ZP/HP) was prepared and functionalized with chitosan (CS@ZP/HP) and β-cyclodextrin (CD@ZP/HP) as potential carriers of 5-Fu. CS@ZP/HP (301.3 mg/g) and CD@ZP/HP (342.8 mg/g) displayed better loading performances than ZP/HP (238.9 mg/g). The higher density of loading sites of CS@ZP/HP (93.87 mg/g) and CD@ZP/HP (117.8 mg/g) than ZP/HP (78.85 mg/g) and enhancement in the organic affinities and surface area illustrate that. The loading of 5-Fu is regulated by multi-molecular and physical mechanistic steps considering the loading (<40 kJ/mol) and Gaussian (<8 kJ/mol) energies. The composites showed slow release profiles (120 h at pH 1.2 and 60 h at pH 7.4) but were still faster than ZP/HP. Both diffusion and erosion processes cooperated during the release of 5-Fu based on the kinetic investigation. The anti-cancer performance of 5-Fu against the HCT-116 colorectal cancer cell lines was enhanced at a remarkable rate after its loading into ZP/HP, CS@ZP/HP, and CD@ZP/HP in order.

## Data Availability

The original contributions presented in the study are included in the article/Supplementary Material, further inquiries can be directed to the corresponding authors.
